# Ice recrystallisation inhibiting polymer nano-objects *via* saline-tolerant polymerisation-induced self-assembly†

**DOI:** 10.1039/D0MH00354A

**Published:** 2020-05-14

**Authors:** Panagiotis G. Georgiou, Ioanna Kontopoulou, Thomas R. Congdon, Matthew I. Gibson

**Affiliations:** a Department of Chemistry, University of Warwick, CV4 7AL, UK; b Warwick Medical School, University of Warwick, CV4 7AL, UK

## Abstract

Chemical tools to modulate ice formation/growth have great (bio)-technological value, with ice binding/antifreeze proteins being exciting targets for biomimetic materials. Here we introduce polymer nanomaterials that are potent inhibitors of ice recrystallisation using polymerisation-induced self-assembly (PISA), employing a poly(vinyl alcohol) graft macromolecular chain transfer agent (macro-CTA). Crucially, engineering the core-forming block with diacetone acrylamide enabled PISA to be conducted in saline, whereas poly(2-hydroxypropyl methacrylate) cores led to coagulation. The most active particles inhibited ice growth as low as 0.5 mg mL^–1^, and were more active than the PVA stabiliser block alone, showing that the dense packing of this nanoparticle format enhanced activity. This provides a unique route towards colloids capable of modulating ice growth.

Ice formation and growth presents major challenges in the maintenance of infrastructure,^1^ adversely affects food texture^2^ and is a key consideration in the storage and transport of cells^3–6^ or vaccines.^7^ Extremophile organisms, which live in the coldest habitats make use of anti-freeze/ice-binding proteins^8^,^9^ (AFP/IBPs), ice-adhesion proteins^10^ as well as ice-nucleating proteins.^11^ It has emerged that it is possible to reproduce the desirable properties of these diverse proteins using materials science, including polymers (especially poly(vinyl alcohol), PVA)^12–15^ self-assembled small molecules,^16^ graphenics^17,18^ and minerals.^19^ One particularly desirable property is ice recrystallisation inhibition (IRI); the prevention of ice crystals from growing (distinct from nucleation). IRI-active materials have been found to improve post-thaw recovery of cryopreserved cells,^17,20,21^ proteins^22^ and bacteria.^23^ It is still not possible to rationally design new IRIs, and in particular generating IRI-active polymer colloids/nanomaterials is challenging. Immobilisation of PVA onto gold particles by ‘grafting to’ retains, but does not enhance, activity^24^ as do coacervates.^25^ Similarly, assembly of Type III AFPs into dendrimers^26^ or on gold particles^27^ allows binding of multiple ice faces, but affords no overall increase in activity. This is surprising as AFP/IBPs typically show more activity as molecular weight or aggregation increases^28,29^ and theory predicts that the rate of ice nucleation requires large protein aggregates to function.^30^ Graphene oxide nanosheets also show strong particle size effects on ice formation.^31^ How large particles can bind and inhibit ice is not clear and if the design rules are related to that of protein/polymers is unknown. There is a clear opportunity to develop nanomaterials containing ice binding macromolecules with controlled presentation and architecture.

Polymerisation-induced self-assembly (PISA) has emerged as a powerful tool to generate multivalent polymeric nanomaterials of controlled morphology and size at high solid contents.^32^ The versatility of this method has allowed the synthesis of nano-objects for drug delivery,^33,34^ cell storage,^35,36^ permeable nano-reactors,^37^,^38^ and has been extensively reviewed.^39–42^ A challenge with many PISA formulations is the need to conduct PISA in biological media, which invariably contain salts. Armes and co-workers engineered stable nanoparticles by changing the steric stabilizer block to poly(sulfobetaines), to enhance solubility in the presence of salt.^43^ Cationic diblock spherical copolymer nanoparticles have also shown surprisingly strong aggregation resistance in electrolyte solutions.^44^ However, if PISA is to be used to design nanoparticles to interface and modulate the growth of ice, the corona-forming block is limited to polymers which can bind ice (*e.g.* PVA).

Taking into account the above, we here address the challenges of (i) conducting PISA in saline without restricting the corona-forming block, and (ii) introducing ice recrystallisation inhibition activity into polymer particles. This is achieved using diacetone acrylamide, rather than the common 2-hydroxypropyl methacrylate coreforming monomer, with a poly(vinyl alcohol) based macrograft-RAFT agent. The resulting particles are potent IRI agents and present a unique approach to employ self-assembled colloidal dispersions for IRI.

Poly(vinyl alcohol) was selected as the corona-forming block, as it is the most active polymeric IRI agent known.^45^ Commercially available PVA (10 kDa/DP = 181) was selected which was grafted with 4-cyano-4-[(ethylsulfanylthiocarbonyl)sulfanyl]pentanoic acid CTA (CEPA), by DCC coupling to afford a water soluble PVA-based graft macro-CTA. The macro-CTA was thoroughly dialysed to remove unfunctionalised RAFT agent. Due to overlapping signals, quantification of the grafting density by ^1^H NMR spectroscopy was not possible. Thermogravimetric analysis (TGA) showed that a ratio of four CEPA molecules per a hundred hydroxyl groups were present, meaning that approximately seven CTA groups were grafted per PVA chain (ESI†). SEC analysis of PVA_181_ graft macro-CTA revealed a small increase in molecular weight compared to PVA_181_ (ESI†).

The most widely used core-forming monomer for PISA, 2-hydroxypropyl methacrylate (HPMA), was used for thermally-initiated dispersion polymerisations at 60 °C (10% w/w) in water, and later in 0.01 M saline. NaCl is essential for IRI activity testing and lack of saline dispersibility would prevent assessment of activity.^46^ In water, monomer conversions above 95% (as confirmed by ^1^H NMR analysis) and stable PISA dispersions were obtained when targeting PHPMA degrees of polymerisation of 50–300. Dynamic light scattering revealed nanoparticles ranging in diameter from 200–400 nm as a function of PHPMA chain length. Dry-state TEM (Fig. 1C) revealed the formation of only spherical morphologies (see ESI† for all characterisation) and this was confirmed by cryo-TEM for PVA_181_-g^7^-PHPMA_50_ (Fig. 1D). We hypothesise that the graft nature of the macro-initiator, along with the high-molecular weight stabilising PVA chain, favours the formation of kinetically-trapped spherical morphologies,^47–51^ but further investigation is beyond the scope of this work. Attempts at dispersing these particles into NaCl (0.1 to 0.01 M) led to macroscopic precipitation. Alternatively, PISA was attempted directly in saline solutions, but no stable particles could be obtained. Therefore, this formulation, despite containing the desired PVA corona, could not be used for IRI activity measurements.^45^ The intolerance of many monomers used in PISA to saline solutions, specifically their inability to self-assemble and tendency to precipitate in such polar media, is a common challenge with PISA formulations and therefore saline-free buffers are often used.^38^

It is not possible to change the PVA corona for another polymer as the PVA is the crucial ice-binding unit. To overcome the saline instability challenge, we therefore reasoned that tuning the core-forming block may enable saline-tolerant PISA. Diacetone acrylamide (DAAm) was investigated as an alternative. In contrast to HPMA, the DAAm reactions (using the same polymerisation conditions as above) led to stable nanoparticle dispersions in both water and in 0.01 M NaCl, with high conversions achieved (>97%). It should be noted that we conducted the screening of PISA in saline, to ensure we selected stable particles, and to avoid problems due to osmotic pressures upon changing [NaCl], but we anticipate that aqueous synthesis followed by addition of saline could also be used. DLS and TEM analysis (Fig. 2) confirmed the formation of spheres in all cases, agreeing with our hypothesis (from the HPMA system) that the graft macro RAFT agent constrains morphology evolution. (We stress our aim here was to probe the IRI activity and not to explore the phase behaviour.) Cryo-TEM imaging on particles also confirmed the formation of spheres (ESI†).

To evaluate the ice recrystallisation inhibition activity, the ‘splat’ assay was used.^13^,^46^ It is important to note that this assay requires saline to create channels between ice crystals, enabling recrystallisation to readily occur and hence avoid false positive results. For this reason the importance of our saline-stabilised PISA system cannot be understated. Polynucleated ice wafers were obtained from 10 μL droplets at –80 °C, then annealed at –8 °C for 30 minutes before being photographed and the average crystal size determined. This method separates nucleation from growth, and lower values of mean grain size (MGS) indicates higher activity. The IRI activity of particles from Fig. 2 are shown in Fig. 3. All the PISA formulations inhibited ice recrystallisation below 5 mg mL^–1^, and in some cases as low as 0.5 mg mL^–1^, placing them in the highly to very highly active range of antifreeze-protein mimetics.^45^ To enable comparison of multivalent effects (as the particles have different sizes) Fig. 3C shows the data in terms of total PVA concentration. At 0.5 mg mL^–1^ PVA_181_-g^7^-PDAAm_50_ showed IRI activity (MGS 40%) but as the core block length increased to PVA_181_-g^7^-PDAAm_300_ there was an increase with larger particles inhibiting growth at 0.1 mg mL^–1^ PVA. This activity, in terms of [PVA] was greater than the macro-CTA alone, implying a multivalent enhancement. Previous reports of PVA and antifreeze proteins grafted to particles or dendrimers^24–27^ gave no relative enhancement. We hypothesise that the dense packing of polymer chains in a PISA particle, compared to those obtained by ‘grafting to’ is crucial to enhance the IRI activity. It is important to note that the macro-CTA had lower IRI activity than pure PVA homopolymer. We used commercial PVA (~80% hydrolysis), which along with the grafted RAFT agent, breaks up the consecutive hydroxyl group sequence, which has been shown to be crucial for PVA IRI.^13^,^52^,^53^

The scaling behaviour of IRI’s in different salt solutions may also affect the magnitude of the results seen here.^54^ To further demonstrate the IRI activity, an alternative assay was conducted in 45 wt% sucrose (‘sucrose sandwich’).^26,55^ Fig. 3D shows nucleated ice crystals, which after 2 hours have grown significantly (recrystallised) in the absence of particles, Fig. 3E. Fig. 3F shows that solutions containing 1 mg mL^–1^ of PVA_181_-g^7^-PDAAm_300_ display complete inhibition of ice recrystallisation, demonstrating activity in a range of different formulations. Finally, ice morphology analysis was conducted. In these assays the temperature is varied to encourage crystal growth and to visualise any morphology changes. PVA is known to bind prismatic faces and hence produces faceting (Fig. 3G) compared to the solution alone (Fig. 3H), suggesting binding is occurring.^15^

## Conclusions

In summary, we present a new concept in the design of biomimetics for controlling ice growth, based upon polymer nanoparticles with densely grafted coronas. Polymerisation-induced self-assembly, PISA, was used as a scalable and tuneable tool to obtain polymer particles from a PVA-graft macroinitiator. By using DAAm as the core-forming monomer, it was possible to conduct PISA directly in saline solution, compared to using HPMA which led to macroscopic coagulation. Using this system, spherical nanoparticles ranging from 200-400 nm were obtained and all were found to be capable of inhibiting ice recrystallisation. The larger particles were found to be more active than the smaller, and ice shaping analysis confirmed binding. These results show that it is possible to develop polymer particles capable of modulating ice growth processes, which may find application from biomedical to infrastructure challenges where ice is a problem. It also offers a practical solution to obtain saline-stable PISA assemblies.

## Supplementary Material

† Electronic supplementary information (ESI) available: This includes full synthetic methods and additional characterisation.

Supporting Information

## Figures and Tables

**Fig. 1:**
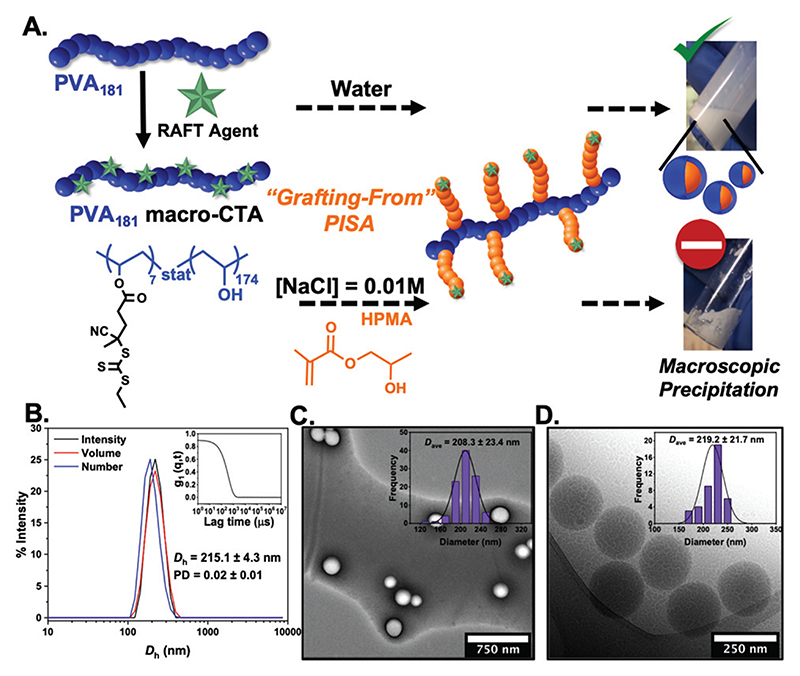
(A) Schematic of the synthetic route for PVA_181_-g^7^-PHPMA_*n*_ nano-objects at 10% w/w *via* thermally initiated RAFT dispersion PISA at 60 °C, using a PVA_181_ macro-CTA. Photographs show precipitation in saline solution and stable dispersions in water. Characterisation of PVA_181_-g^7^-PHPMA_50_ nano-objects in aqueous solution; (B) intensity-weighted size distributions, average *D_h_* and PD values from DLS (error from *n* = 5). Inset: Autocorrelation function; representative dry-state (C) and cryo-TEM (D) images (insets show size distribution histograms along with average diameter; *n* > 50).

**Fig. 2:**
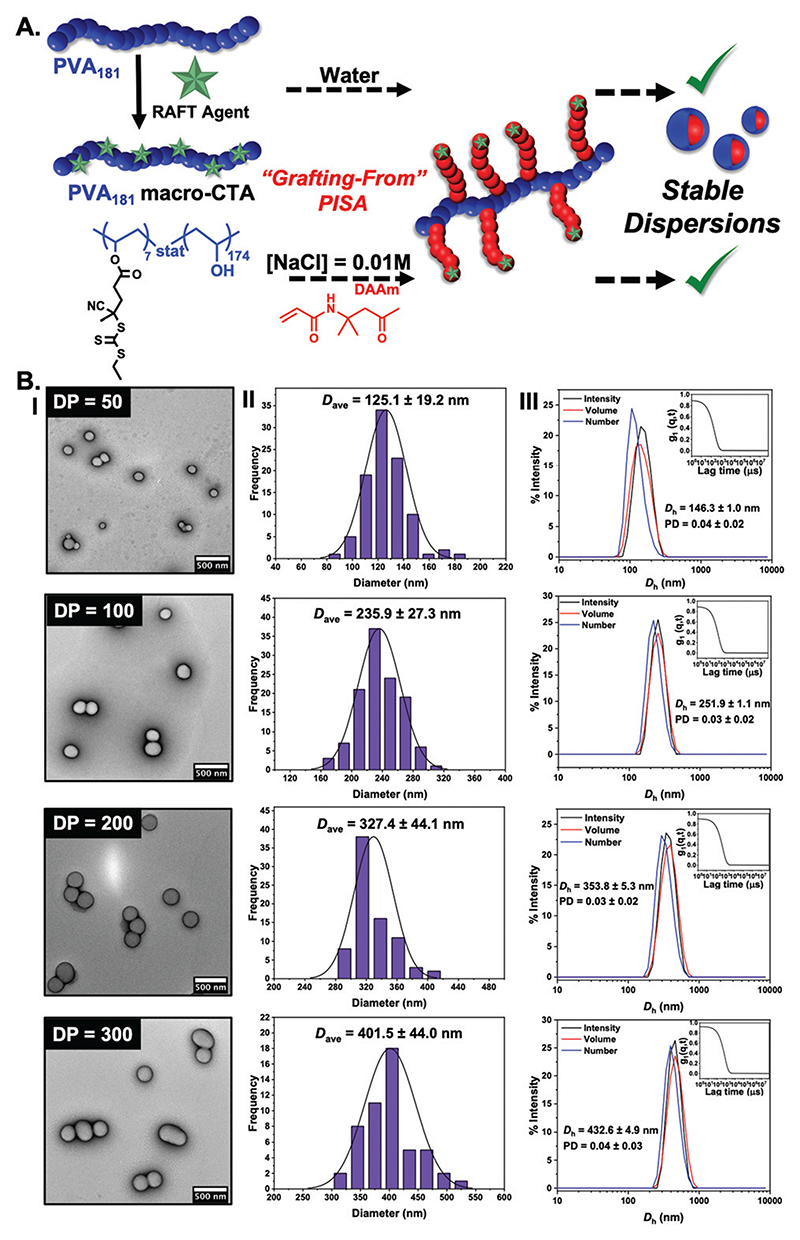
(A) Schematic of the synthetic route employed for the preparation of PVA_181_-g^7^-PDAAm_*n*_ (*n* = 50,100, 200, 300) nano-objects at 10% w/w *via* thermally initiated RAFT dispersion PISA at 60 °C, using a PVA_181_ macro-CTA resulting in stable dispersion in both water and in saline; (B) characterisation of PDAAm-core nanoparticles as a function of PDAAm degree of polymerization by TEM (I), showing the particle size distribution (II) and also by DLS (III). See ESI† for cryo-TEM analysis. Histograms are from n > 50, and DLS is averaged from *n* = 5.

**Fig. 3:**
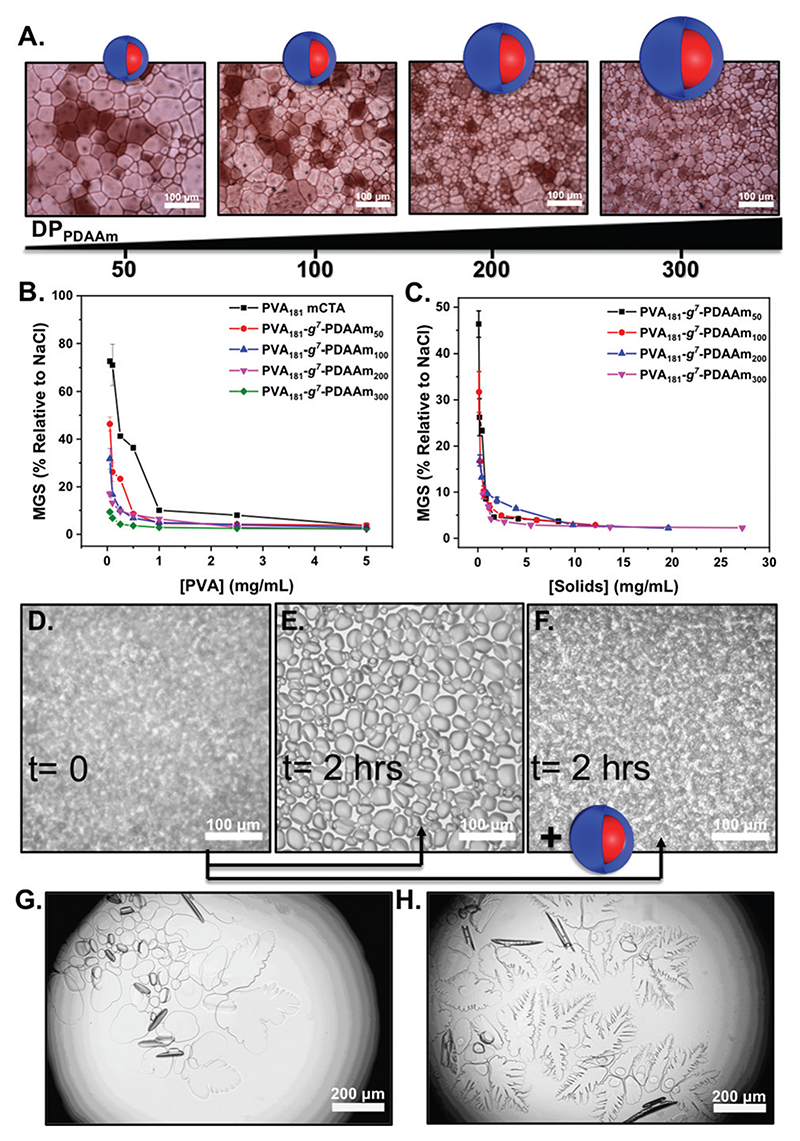
Assessment of IRI and ice binding activity. (A) Cryomicrographs from the ‘splat’ assay of nanoparticles. [PVA] = 0.05 mg mL^-1^; (B) IRI activity corrected to [PVA]; (C) IRI activity in mass concentration; (D) ice growth in 45 wt% sucrose *t* = 0; (E) *t* =2 hours, (F) *t* = 2 hours plus 1 mg mL^-1^ of PVA_181_-g^7^-PDAAm_300_ nanoparticles; (G) ice shaping of sucrose solution alone; (H), 1 mg mL^-1^ of PVA_181_-g^7^-PDAAm_300_ nanoparticles. MGS = mean grain size.

## New concepts

In this manuscript, the concept of using polymerisation-induced selfassembly (PISA) to generate unique nanomaterials that are capable of inhibiting ice growth is shown for the first time. Ice growth is a major problem in cell storage, infrastructure maintenance and in the food industry. Existing solutions to control ice growth have focussed on using antifreeze/ice-binding proteins from extremophile organisms and more recently polymeric inhibitors have emerged. Previous reports of nanomaterial architectures containing ice recrystallisation active macromolecules did not show enhancements in activity. In contrast native antifreeze/ice-binding proteins show size and aggregation state dependent activity, which we have successfully mimicked here. Crucially to achieve this, we tuned the PISA process to allow it to be conducted in saline solution by careful selection of the core-forming block enabling us to use our desired (and indeed essential) coronal macroinitiator. Secondly, due to the dense surface grafting of the coronal block, the resulting particles are extremely potent IRIs functioning below 1 mg ml. ^-1^. This new ice growth controlling particle formulation opens the door to advanced colloidal dispersions capable of withstanding freeze stress as well as bringing new understanding.
