# Supplementary material for: Ice recrystallisation inhibiting polymer nano-objects via saline-tolerant polymerisation-induced self-assembly†
Source: Mater Horiz. Author manuscript; Available in PMC 2021 Mar 9. (PMC7116880; doi:10.1039/D0MH00354A)
Supplement: Supporting Information [file EMS117806-supplement-Supporting_Information.pdf]

Electronic Supplementary Information

Ice recrystallisation inhibiting polymer nanoparticles from saline-tolerant polymerisation-induced self-assembly

*Panagiotis G. Georgiou,[a] Ioanna Kontopoulou,[a] Thomas Congdon,[a] and Matthew I. Gibson[a,b]**

[a] Department of Chemistry, University of Warwick, Gibbet Hill Road, CV4 7AL, Coventry, UK

[b] Warwick Medical School, University of Warwick, Gibbet Hill Road, CV4 7AL, Coventry, UK

**Corresponding Author*: m.i.gibson@warwick.ac.uk (M.I.G)

Contents

Experimental Section S2

Materials and Methods S2

Materials S2

Characterisation Techniques S2

Experimental Methods S3

Synthetic Methods S5

Supplementary Characterisation Data S9

References S17

Experimental Section

Materials and Methods

Materials

Poly(vinyl alcohol) (average M_n = 9,000-10,000 g.mol^{-1}, 80% hydrolysed, PVA), sodium ethanethiolate, carbon disulfide (anhydrous, ≥99%), *N*,*N*′-dicyclohexylcarbodiimide (99%, DCC), 4-(dimethylamino)pyridine (≥98%, DMAP), 2,2′-azobis(2-methylpropionamidine) dihydrochloride (97%, VAZO-50), sucrose (≥99.5%), dichloromethane (anhydrous, ≥99.8%, DCM), dimethyl sulfoxide (anhydrous, ≥99.9%, DMSO) were purchased from Sigma Aldrich and were used without further purification. Iodine, diethyl ether and dichloromethane (DCM) were purchased from Fisher Scientific. Ethyl acetate was purchased from VWR Chemicals. The monomer 2-hydroxypropyl methacrylate (mixture of isomers, 2-hydroxypropyl methacrylate and 2-hydroxyisopropyl methacrylate, 97%, HPMA) was also purchased from Sigma Aldrich and was passed through a column of basic alumina to remove inhibitor prior to use. Diacetone acrylamide (99%, DAAm) and 4,4'-azobis(4-cyanovaleric acid) (98%, ACVA) were obtained from Alfa Aesar. Sodium chloride (≥99%) was purchased from Fisher Scientific. Dialysis membrane (MWCO = 1000 Da) was purchased from Spectra/Por. Formvar-carbon coated copper grids were purchased from EM Resolutions.

Characterisation Techniques

NMR Spectroscopy. ^{1}H-NMR and ^{13}C-NMR spectra were recorded at 300 MHz or 400 MHz on a Bruker DPX-300 or DPX-400 spectrometer respectively, with chloroform-*d* ($CDCl_3$), methanol-d_4 (CD_3OD) and DMSO-d_6 ($(CD_3)_2SO$) as the solvent. Chemical shifts of protons are reported as δ in parts per million (ppm) and are relative to tetramethylsilane (TMS) at δ = 0 ppm when using $CDCl_3$ or solvent residual peak (CH_3OH, δ = 3.31 ppm/ DMSO, δ = 2.50 ppm).

Size Exclusion Chromatography. Size exclusion chromatography (SEC) analysis was performed on an Agilent Infinity II MDS instrument equipped with differential refractive index (DRI), viscometry (VS), dual angle light scatter (LS) and variable wavelength UV detectors. The system was equipped with 2 x PLgel Mixed D columns (300 x 7.5 mm) and a PLgel 5 µm guard column. The mobile phase used was DMF (HPLC grade) containing 5 mM NH_4BF_4 at 50 °C at flow rate of 1.0 mL.min^{-1}. Poly(methyl methacrylate) (PMMA) standards (Agilent EasyVials) were used for calibration between 955,000 – 550 g.mol^{-1}. Analyte samples were filtered through a nylon membrane with 0.22 μm pore size before injection. Number average molecular weights (M_n), weight average molecular weights (M_w) and dispersities ($Đ_M = M_w/M_n$) were determined by conventional calibration and universal calibration using Agilent GPC/SEC software.

Thermogravimetric (TGA) analysis. TGA was performed on a Mettler-Toledo Star^{e} TGA/DSC instrument with autosampler. Measurements were carried out under a nitrogen atmosphere from 50 to 700 °C at a rate of 5 °C min^{-1} using a standard 70 μL alumina crucibles.

Dynamic Light Scattering. Hydrodynamic diameters (D_h) and size distributions of particles were determined by dynamic light scattering (DLS) using a Malvern Zetasizer Nano ZS with a 4 mW He-Ne 633 nm laser module operating at 25 °C. Measurements were carried out at an angle of 173° (back scattering), and results were analysed using Malvern DTS 7.03 software. All determinations were repeated 5 times with at least 10 measurements recorded for each run. D_h values were calculated using the Stokes-Einstein equation where particles are assumed to be spherical.

Zeta Potential Analysis. Zeta potential was measured by the technique of microelectrophoresis, using a Malvern Zetasizer Nano ZS instrument, at room temperature at 633 nm. All reported measurements were the average of at least five runs. Zeta potential was calculated from the corresponding electrophoretic mobilities (μ_E) by using the Henry's correction of the Smoluchowski equation ($\mu_E = 4\pi \varepsilon_0 \varepsilon_r \zeta (1+\kappa r)/6\pi \mu$).

Transmission Electron Microscopy. Dry-state stained TEM imaging was performed on either a JEOL JEM-2100 or a JEOL JEM-2100Plus microscope operating at an acceleration voltage of 200 kV. All dry-state samples were diluted with MilliQ water and then deposited onto formvar-coated copper grids. After roughly 1 min, excess sample was blotted from the grid and the grid was stained with an aqueous 1 wt% uranyl acetate (UA) solution for 1 min prior to blotting, drying and microscopic analysis.
Cryogenic transmission electron microscopy (cryo-TEM) imaging was performed on a JEOL JEM-2100Plus microscope operating at an acceleration voltage of 200 kV. Samples for cryo-TEM imaging were prepared at 0.5% w/w solids content in milliQ water by depositing 8 μL sample onto plasma-treated lacey-carbon coated grids followed by blotting for approximately 5 s and plunging into a pool of liquid ethane, cooled using liquid nitrogen, to vitrify the samples. Transfer into a pre-cooled cryo-TEM holder was performed under liquid nitrogen temperatures prior to microscopic analysis. For the determination of average size of spherical assemblies at least 50 particles were analysed in each case.

Experimental Methods

Splat Ice Recrystallisation Inhibition Assay. Splat cooling assays were performed as previously described by Tomczak *et al.*[1] Briefly, a 10 μL sample was dropped 1.40 m onto a chilled glass coverslip, resting on a thin aluminium block placed on dry ice. Upon hitting the coverslip, a wafer with diameter of approximately 10 mm and thickness 10 μm was formed instantaneously. The glass

coverslip was transferred onto the Linkam cryostage and held at -8°C using liquid nitrogen for 30 minutes. Photographs were obtained using an Olympus CX 41 microscope with a UIS-2 20x/0.45/∞/0-2/FN22 lens and crossed polarisers (Olympus Ltd), equipped with a Canon DSLR 500D digital camera. Images were taken of the initial wafer (to ensure that a polycrystalline sample had been obtained) and again after 30 minutes. Image processing was conducted using ImageJ. In brief, the number of ice crystals in the field of view was measured for each photograph. The average (mean) of these three measurements was then calculated to find the mean grain area (MGS). The average value and error were compared to that of [NaCl] = 0.01 M solution, as appropriate, as a negative control. Nanoparticle samples solutions were first prepared upon dilution in [NaCl] = 0.01M on the range of PVA concentrations 5 – 0.05 mg.mL^{-1}.

Sucrose Sandwich Ice Recrystallisation Inhibition Assay. Sucrose sandwich IRI assays were performed as described by Smallwood *et al*.[2,3] Briefly, nano-object samples dispersed in NaCl ([NaCl] = 0.01M) containing 45 wt % sucrose were sandwiched between two coverslips, and the edges were sealed with grease. Samples were cooled to and held at −50°C for 2 min on a Linkam Biological Cryostage BCS196 with T95-Linkpad system controller equipped with a LNP95-Liquid nitrogen cooling pump, using liquid nitrogen as the coolant (Linkam Scientific Instruments UK). The temperature was then elevated to −8°C and held for 2 h. During this time, images were recorded every 10 mins using an Olympus CX41 microscope equipped with a UIS-2 20x/0.45/∞/0−2/FN22 lens (Olympus Ltd.) and a Canon EOS 500D SLR digital. Image processing was conducted using ImageJ.

Modified Sucrose Sandwich Ice Shaping Assay. Nano-object samples dispersed in NaCl ([NaCl] = 0.01M) containing 45 wt % sucrose were sandwiched between two glass coverslips and sealed with immersion oil. Samples were cooled to −50 °C on a Linkam Biological Cryostage BCS196 with T95-Linkpad system controller equipped with a LNP95-Liquid nitrogen cooling pump, using liquid nitrogen as the coolant (Linkam Scientific Instruments UK). The temperature was then increased to −8 °C and held for 1 h to anneal. The samples were then heated at 0.5 °C.min^{-1} until few ice crystals remained and then cooled at 0.05 °C.min^{-1} and the shape of ice crystals observed. Micrographs were obtained every 0.1 °C using an Olympus CX41 microscope equipped with a UIS-2 20x/0.45/∞/0−2/FN22 lens (Olympus Ltd.) and a Canon EOS 500D SLR digital. Image processing was conducted using ImageJ.

Synthetic Methods

Synthesis of 4-cyano-4-[(ethylsulfanylthiocarbonyl)sulfanyl] pentanoic acid (CEPA)

4-Cyano-4-[(ethylsulfanylthiocarbonyl)sulfanyl] pentanoic acid chain transfer agent (CEPA CTA) was synthesised according to a previously described process.[4] In particular, sodium ethanethiolate (5.0 g, 0.59.5 mmol, 1eq) was suspended in 250 mL of dry diethyl ether at 0 °C. Carbon disulfide (3.94 mL, 65.5 mmol, 1.1 eq) was subsequently added dropwise over 10 min, resulting to the formation of a thick yellow precipitate of sodium *S*-ethyl trithiocarbonate. After 2 h of stirring at room temperature, solid iodine (7.49 g, 29.5 mmol, 0.5 eq) was added to the reaction medium. After 2 h, the solution was washed three times with aqueous sodium thiosulfate (1 M, 200 mL), water (200 mL) and finally saturated NaCl solution (200 mL). The organic layer was thoroughly dried over $MgSO_4$ and the crude bis-(ethylsulfanylthiocarbonyl) disulfide was then isolated by rotary evaporation (8.1 g, 29.5 mmol). A solution of bis-(ethylsulfanylthiocarbonyl) disulfide (8.1 g, 29.5 mmol, 1 eq) and 4,4′-azobis(4-cyanopentanoic acid) (ACVA) (12.4 g, 44.25 mmol, 1.5 eq) in 250 mL ethyl acetate was heated at reflux for 18 h under N_2 (g) atmosphere. Following rotary evaporation of the solvent, the crude CEPA CTA was isolated by flash column chromatography using silica gel as the stationary phase and 75:25 DCM-petroleum ether 60-80 °C as the eluent. The isolated product was precipitated out of solution using hexane, leaving a yellow-light orange solid. The final product was collected and dried under reduced pressure to afford pure CEPA CTA (7.42 g, 58.7 mmol, 48%). ^{1}H-NMR (400 MHz, $CDCl_3$): δ (ppm) 3.36 (q, 2H, S-C$\boldsymbol{H_2}$-CH_3), 2.39-2.71 (m, 4H, C$\boldsymbol{H_2}$-C$\boldsymbol{H_2}$), 1.89 (s, 3H, C(CN)-C$\boldsymbol{H_3}$), 1.36 (t, 3H, S-CH_2-C$\boldsymbol{H_3}$). ^{13}C-NMR (100 MHz, $CDCl_3$): δ (ppm) 216.7, 177.0, 118.9, 46.2, 33.5, 31.4, 29.5, 24.7, 12.8. FT-IR (neat): ν (cm^{-1}) 2235 (C≡N), 1709 (C=O), 1073 (C=S).

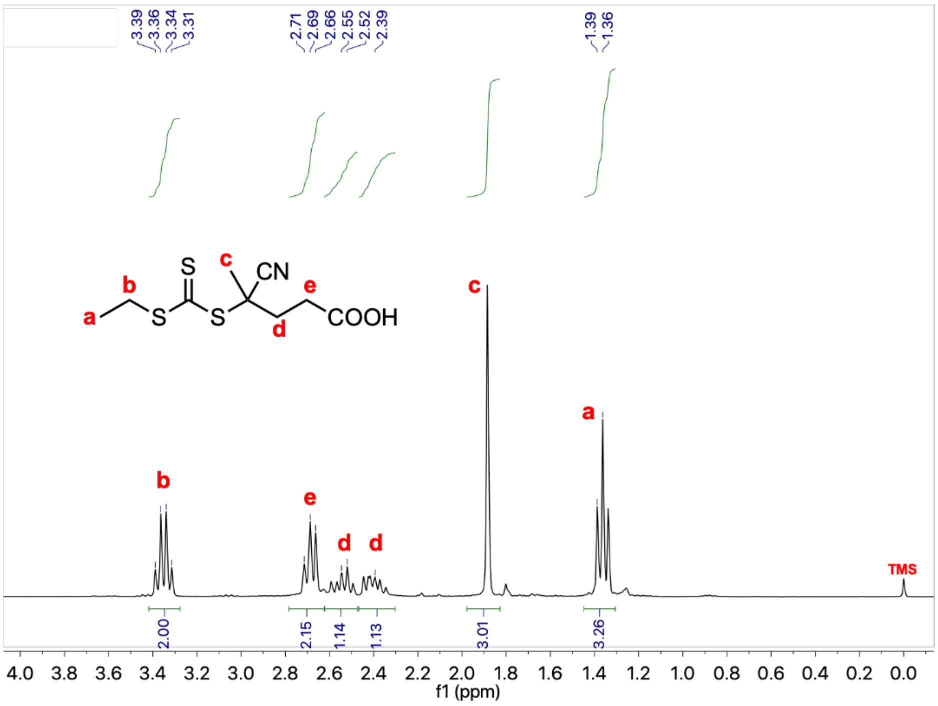

Figure S1. ^{1}H NMR spectrum of 4-cyano-4-[(ethylsulfanylthiocarbonyl)sulfanyl] pentanoic acid in $CDCl_3$.

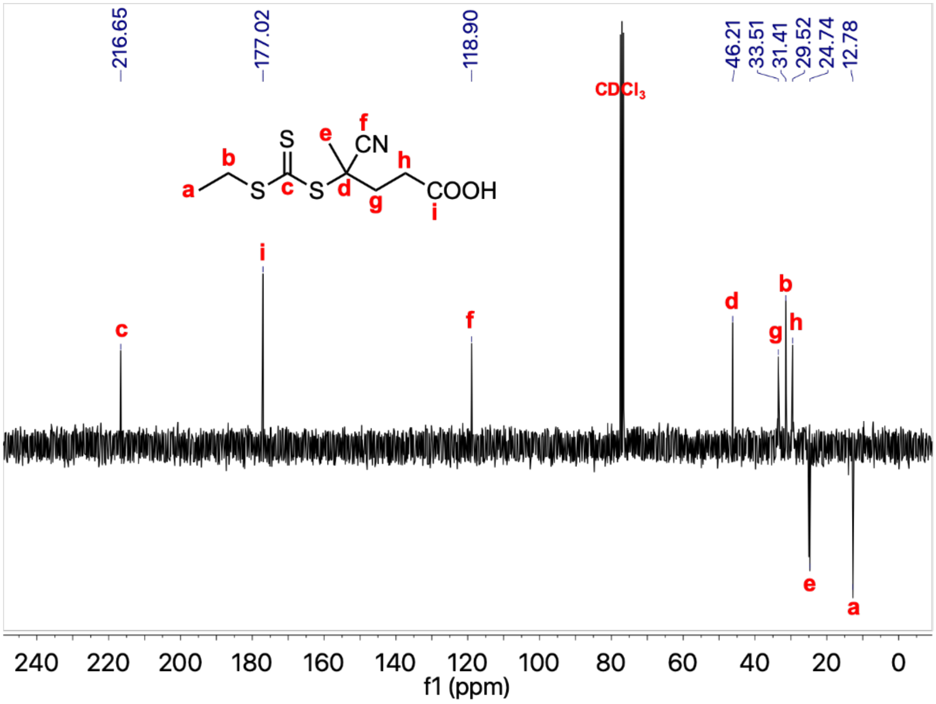

Figure S2. ^{13}C NMR spectrum of 4-cyano-4-[(ethylsulfanylthiocarbonyl)sulfanyl] pentanoic acid in $CDCl_3$.

Synthesis of poly(vinyl alcohol)$_{181}$-CEPA graft macro-CTA (PVA$_{181}$-CEPA macro-CTA). PVA$_{181}$ containing 7 CEPA groups per poly(vinyl alcohol) chain, was prepared *via* partial coupling esterification of the hydroxyl groups of poly(vinyl alcohol) with carboxylic acid-end group of CEPA in the presence of dicyclohexylcarbodiimide (DCC) according to previously reported methods.[5,6] Poly(vinyl alcohol) (M_n ~ 9,000 g mol^{-1}, PVA$_{181}$, 80% hydrolysed) (1 g, approx. 1.25×10^{-4} mol of vinyl alcohol units, 1 eq) was dissolved in 25 mL of dry DMSO. The resulting solution was then purged with N_2 (g) for 30 min. After complete dissolution, CEPA CTA (0.329 g, 1.25×10^{-3} mol, 10 eq), DCC (51.6 mg, 2.50×10^{-4} mol, 2 eq) and DMAP (3.1 mg, 2.5×10^{-5} mol, 0.2 eq) were added to the reaction mixture. The esterification reaction proceeded with stirring at room temperature for 18 h under continuous N_2 (g) flow. After this period, solution was filtered to remove unreacted DCC and DMAP. The resulted PVA$_{181}$-CEPA graft macro-CTA was collected by precipitation into 250 mL of cold ethyl acetate, redissolved in milliQ water and dialysed against water for 48 h (dialysis membrane MWCO = 1000 Da). The purified PVA$_{181}$-CEPA graft macro-CTA was then lyophilised to give a light yellow solid as the final product (0.87 g, 8.0×10^{-5} mol, 71%) and then analysed by ^{1}H NMR, DMF SEC (Figure S2). Due to overlapping signals it was unable to determine exact CTA units per polymer chain through ^{1}H NMR analysis. Therefore thermogravimetric (TGA) analysis was employed (Figure S1). ^{1}H NMR (400 MHz, DMSO-d_6): δ (ppm) 5.15-4.05 (br m, CH-O***H*** of PVA), 3.83 (s, C***H***-OH of PVA backbone), 3.62 (s, CH$_2$-C***H***O of PVAc backbone), 3.17 (q, 2H, CH$_3$-C$\boldsymbol{H_2}$-S-(C=S)), 2.77 (m, 2H, C$\boldsymbol{H_2}$-CO$_2$), 2.31 (t, 3H, C$\boldsymbol{H_3}$-CH$_2$-S-(C=S)), 1.95 (C$\boldsymbol{H_2}$-CHO of PVAc backbone), 1.66-1.13 (br m, CH-C$\boldsymbol{H_2}$ of PVA backbone). SEC (5 mM NH_4BF_4 in DMF) $M_{n,\,SEC\,RI}$ = 29,300 g mol^{-1}, $Đ_{M\,RI}$ = 1.71.

Synthesis of PVA$_{181}$-g^7-PHPMA$_n$ based nano-objects by aqueous RAFT-mediated dispersion thermally-initiated polymerisation-induced self-assembly (PISA). A typical synthetic procedure to achieve PVA$_{181}$-g^7-PHPMA$_{200}$ nano-objects at 10 wt% solids content by aqueous RAFT-mediated PISA is described. To a 6 mL vial containing a stirring bar were added PVA$_{181}$ macro-CTA (40 mg, 3.7×10^{-6} mol, 1 eq), HPMA (106 mg, 7.4×10^{-4} mol, 200 eq), VAZO-50 (0.05 mg, 1.84×10^{-7} mol, 0.05 eq) and MiliQ H_2O (1.32 mL) such that the final concentration of monomer was 10 % w/w. The vial was sealed with a rubber septum and the solution was then purged with N_2(g) for 20 min. The vial was placed into an aluminium heating block which had been pre-heated to 60 °C. After 4 h, the polymerisation was quenched by cooling the reaction mixture to room temperature and exposing it to air. An aliquot was removed for ^{1}H NMR in methanol-d_4 and DMF SEC analyses. The resulting solution of particles was purified by three centrifugation/resuspension cycles in milliQ water at 14000 rpm. TEM, DLS and zeta potential analyses were performed on samples after dilution to an appropriate analysis concentration.

Synthesis of PVA_{181}-g^7-$PDAAm_n$ based nano-objects by saline RAFT-mediated dispersion thermally-initiated polymerisation-induced self-assembly (PISA). A typical synthetic procedure to achieve PVA_{181}-g^7-$PDAAm_{200}$ nano-objects at 10 wt% solids content by RAFT-mediated PISA in saline medium is described. To a 6 mL vial containing a stirring bar were added PVA_{181} macro-CTA (40 mg, 3.7×10^{-6} mol, 1 eq), DAAm (125 mg, 7.4×10^{-4} mol, 200 eq), VAZO-50 (0.05 mg, 1.84×10^{-7} mol, 0.05 eq) and NaCl solution (0.01M) (1.48 mL) such that the final concentration of monomer was 10% w/w. The vial was sealed with a rubber septum and the solution was then purged with N_2 (g) for 20 min. The vial was placed into an aluminium heating block which had been pre-heated to 60 °C. After 4 h, the polymerisation was quenched by cooling the reaction mixture to room temperature and exposing it to air. An aliquot was removed for ^{1}H NMR in methanol-d_4 and DMF SEC analyses. The resulting solution of particles was purified by three centrifugation/resuspension cycles in NaCl solution (0.01 M) at 14000 rpm. TEM, DLS and zeta potential analyses were performed on samples after dilution to an appropriate analysis concentration.

Supplementary Characterisation Data

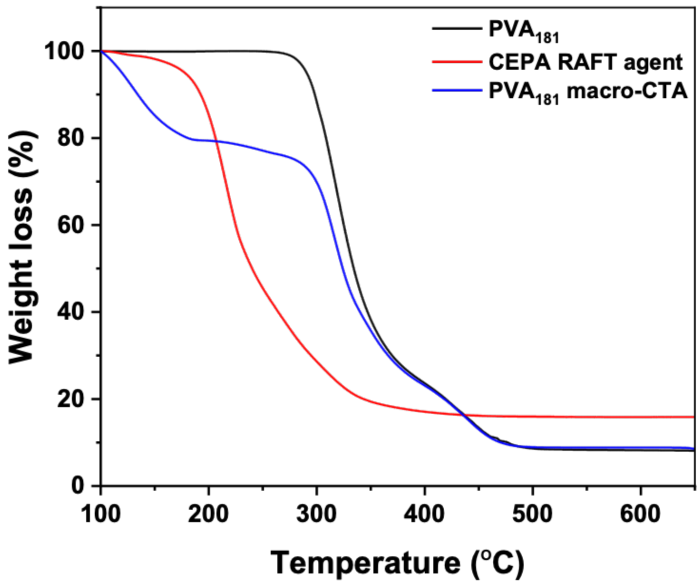

Figure S1. Thermogravimetric analysis (TGA) of polyvinyl alcohol before functionalisation (PVA_{181} – black trace), CEPA chain transfer agent (red trace) and polyvinyl alcohol graft macro-CTA (PVA_{181} macro-CTA, blue trace). Based on first transition at 150 ºC, 20% weight loss of total mass is observed which corresponds to the CTA moieties grafted on polymer chain. This equals to approximately 2000 Da (7 units) of CEPA RAFT agent.

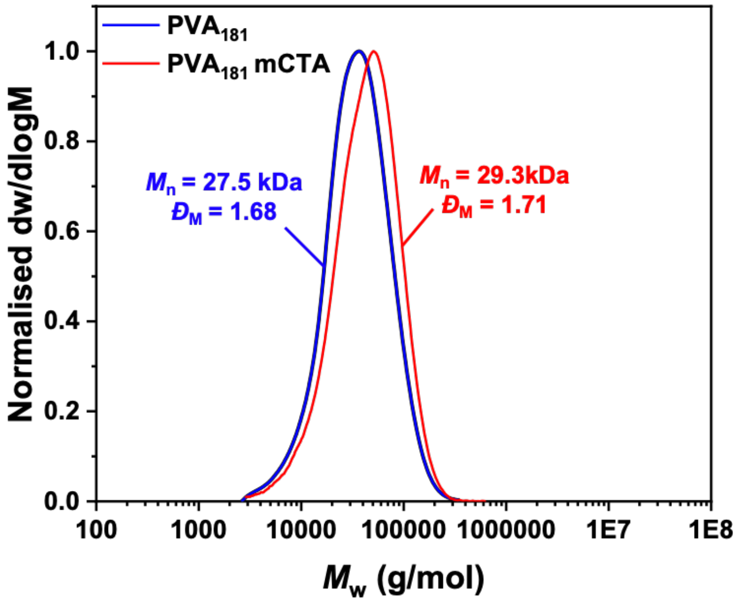

Figure S2. Normalised SEC RI molecular weight distributions for PVA_{181} (blue trace) and PVA_{181} CEPA graft macro-CTA (red trace) along with the corresponding M_n and $Đ_M$ values. M_n and $Đ_M$ values were calculated from PMMA standards using 5 mM NH_4BF_4 in DMF as the eluent.

Table S1. Summary of final monomer conversions, M_n, $Đ_M$, D_h, PD and zeta-potential values for PVA_{181}-g^7-$PHPMA_n$ (n = 50, 100, 200, and 300) after aqueous PISA at [solids] = 10, wt% for 4 hours, as determined by ^{1}H-NMR spectroscopy, SEC, DLS and microelectrophoretic analysis.

Target DP_{PHPMA}	Solids Content (wt%)	Conversion[a] (%)	$M_{n,\ SEC\ RI}$[b] (kg.mol^{-1})	$Đ_M$[b]	D_h (nm)[c]	PD[c]	Zeta-Potential (mV)[d]
**50**	10	>99	34.9	2.5	215.1 ± 4.3	0.02 ± 0.01	-31.5 ± 0.8
**100**	10	>99	35.7	2.3	276.3 ± 4.0	0.09 ± 0.03	-33.7 ± 0.4
**200**	10	98	71.8	5.7	306.2 ± 4.1	0.07 ± 0.02	-34.9 ± 0.8
**300**	10	97	85.1	8.6	388.7 ± 7.1	0.02 ± 0.01	-33.3 ± 0.3

[a] Monomer conversion calculated from ^{1}H-NMR spectroscopy in methanol-*d*4. [b] M_n and $Đ_M$ values calculated from PMMA standards using 5 mM NH_4BF_4 in DMF as the eluent. [c] D_h and PD values measured from DLS analysis. [d] Zeta-potential values measured from microelectrophoretic analysis at pH = 7.0.

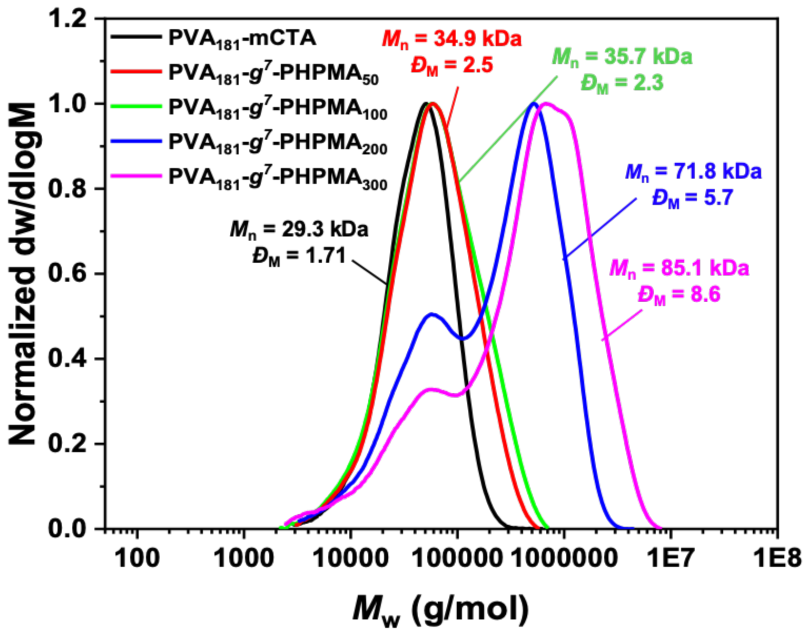

Figure S3. Normalised SEC RI traces for PVA_{181}-CEPA graft macro-CTA (black trace) and PVA_{181}-g^7-$PHPMA_n$ (n = 50-red trace, 100-blue trace, 200-green trace, and 300-purple trace) at [solids] = 10 wt%, using 5 mM NH_4BF_4 in DMF as the eluent.

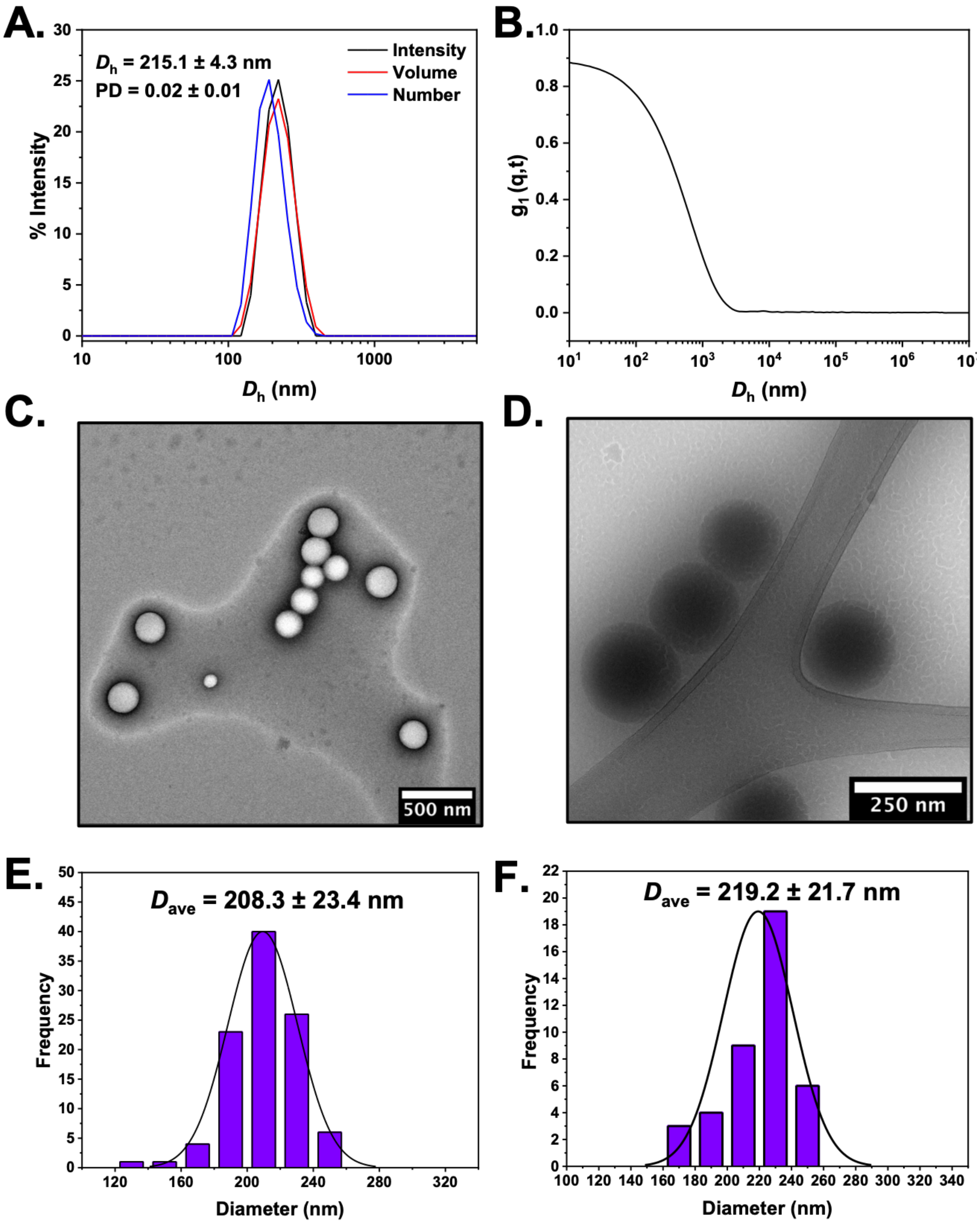

Figure S4. Characterisation of purified PVA$_{181}$-g^{7}-PHPMA$_{50}$ nano-objects. (A) Intensity-weighted size distributions along with average D_h and PD values (the error shows the standard deviation from 5 repeat measurements), and (B) autocorrelation function obtained by DLS. (C) Representative dry-state TEM image, stained with 1 wt% UA solution, and (D) representative cryo-TEM image. Histograms of size distributions along with calculated average diameter, measured from particle analysis based on dry-state TEM (E) and cryo-TEM (F) images. In each case, at least 50 particles were analysed.

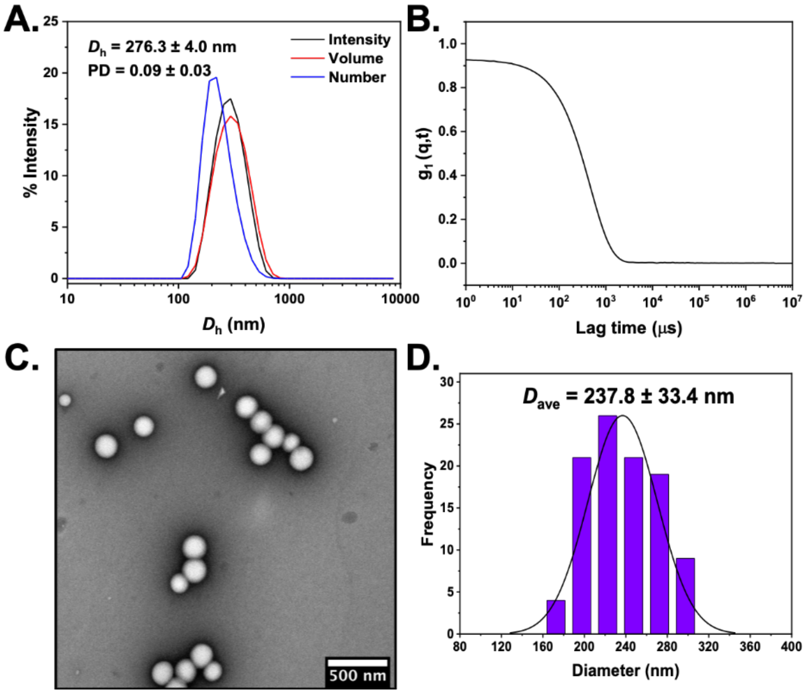

Figure S5. Characterisation of purified PVA$_{181}$-g^7-PHPMA$_{100}$ nano-objects. (A) Intensity-weighted size distributions along with average D_h and PD values (the error shows the standard deviation from 5 repeat measurements), and (B) autocorrelation function obtained by DLS. (C) Representative dry-state TEM image, stained with 1 wt% UA solution, and (D) histogram of size distribution along with calculated average diameter, measured from particle analysis based on dry-state TEM images. In each case, at least 50 particles were analysed.

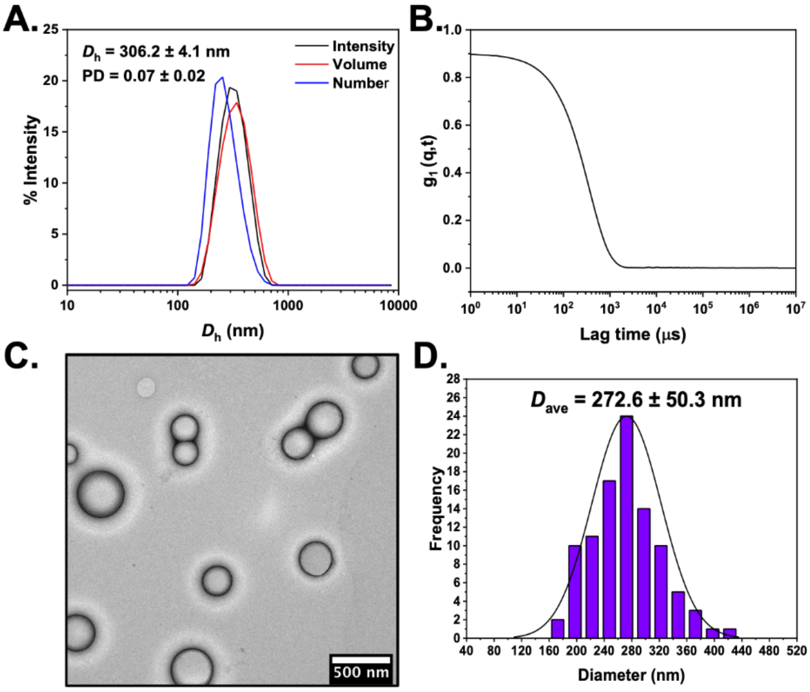

Figure S6. Characterisation of purified PVA$_{181}$-g^7-PHPMA$_{200}$ nano-objects. (A) Intensity-weighted size distributions along with average D_h and PD values (the error shows the standard deviation from 5

repeat measurements), and (B) autocorrelation function obtained by DLS. (C) Representative dry-state TEM image, stained with 1 wt% UA solution, and (D) histogram of size distribution along with calculated average diameter, measured from particle analysis based on dry-state TEM images. In each case, at least 50 particles were analysed.

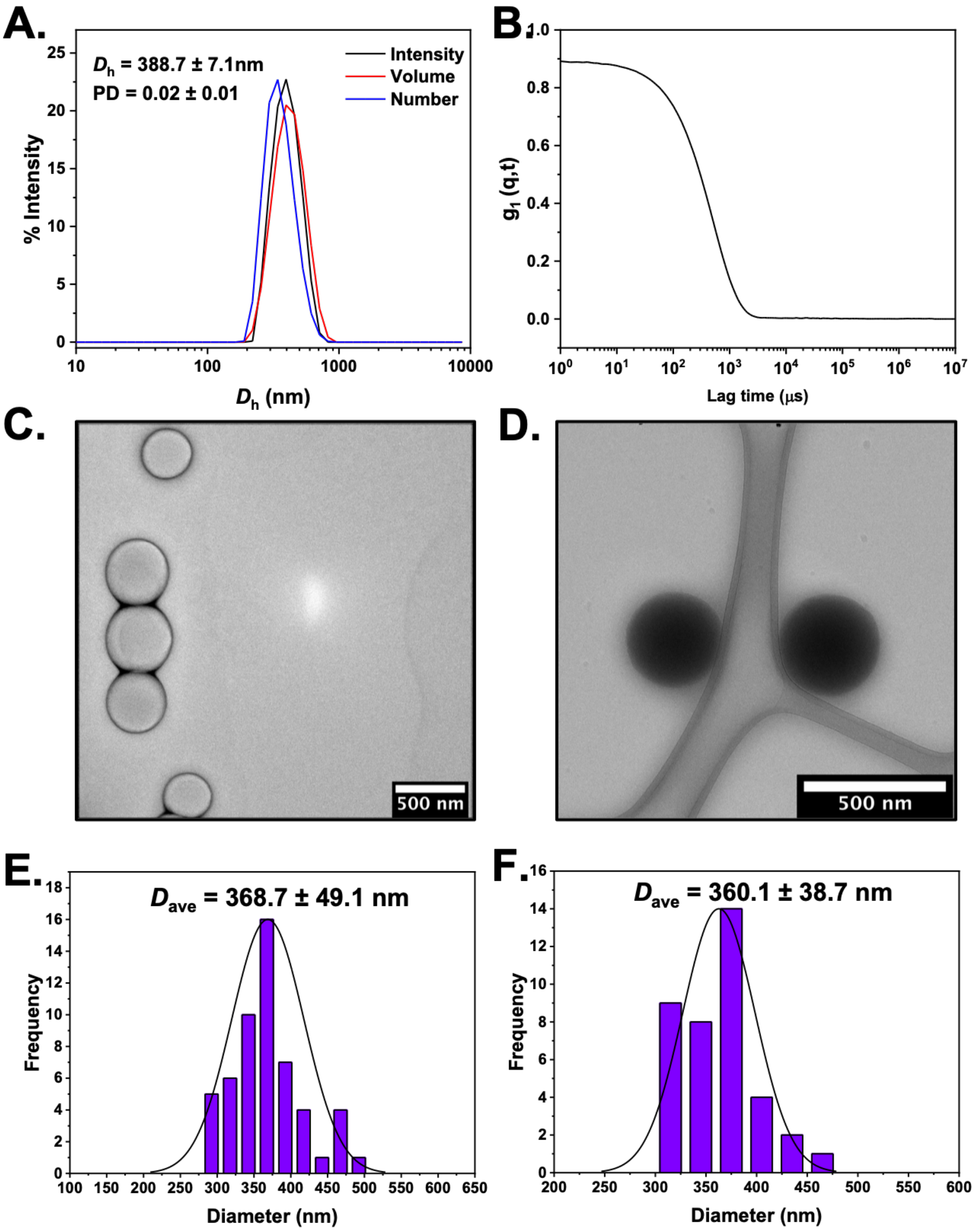

Figure S7. Characterisation of purified PVA_{181}-g^7-$PHPMA_{300}$ nano-objects. (A) Intensity-weighted size distributions along with average D_h and PD values (the error shows the standard deviation from 5 repeat measurements), and (B) autocorrelation function obtained by DLS. (C) Representative dry-state TEM image, stained with 1 wt% UA solution, and (D) representative cryo-TEM image. Histograms of size distributions along with calculated average diameter, measured from particle analysis based on dry-state TEM (E) and cryo-TEM (F) images. In each case, at least 50 particles were analysed.

Table S2. Summary of final monomer conversions, M_n, $Đ_M$, D_h, PD and zeta-potential values for PVA_{181}-g[7]-$PDAAm_n$ (n = 50, 100, 200, and 300) after PISA reactions performed in saline medium ([NaCl] = 0.01 M) at [solids] = 10, wt% for 4 hours, as determined by ^{1}H-NMR spectroscopy, SEC, DLS, microelectrophoretic analysis.

Target DP_{PHPMA}	Solids Content (wt%)	Conversion[a] (%)	$M_{n, SEC RI}$[b] (kg.mol^{-1})	$Đ_M$[b]	D_h (nm)[c]	PD[c]	Zeta-Potential (mV)[d]
**50**	10	>99	44.7	4.3	146.3 ± 1.0	0.04 ± 0.02	-17.1 ± 0.2
**100**	10	>99	53.2	6.4	251.9 ± 1.1	0.03 ± 0.02	-16.3 ± 0.5
**200**	10	>99	73.8	6.8	353.8 ± 5.3	0.03 ± 0.02	-10.3 ± 0.3
**300**	10	>99	114.6	7.1	432.6 ± 4.9	0.04 ± 0.03	-18.7 ± 0.1

[a] Monomer conversion calculated from ^{1}H-NMR spectroscopy in methanol-d_4. [b] M_n and $Đ_M$ values calculated from PMMA standards using 5 mM NH_4BF_4 in DMF as the eluent. [c] D_h and PD values measured from DLS analysis. [d] Zeta-potential values measured from microelectrophoretic analysis at pH = 7.0.

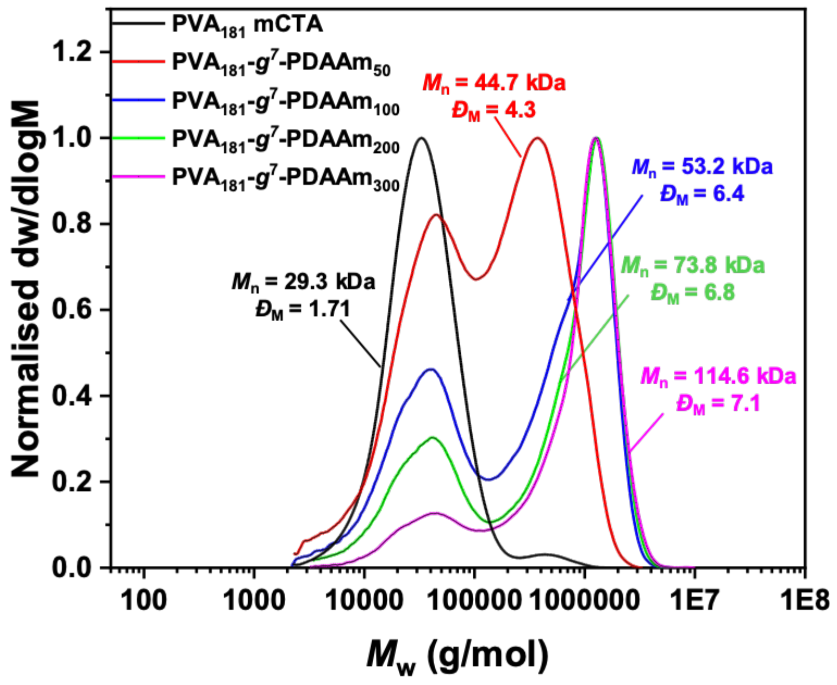

Figure S8. Normalised SEC RI traces for PVA_{181}-CEPA graft macro-CTA (black trace) and PVA_{181}-g[7]-$PDAAm_n$ (n = 50-red trace, 100-blue trace, 200-green trace, and 300-purple trace) at [solids] = 10 wt%, using 5 mM NH_4BF_4 in DMF as the eluent.

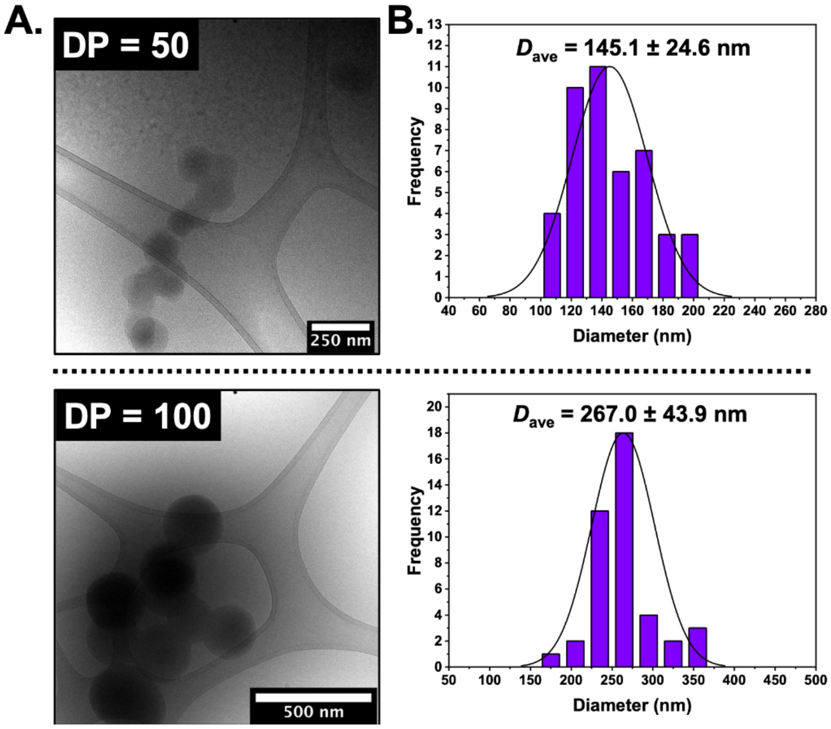

Figure S9. (A) Representative cryo-TEM images for PVA$_{181}$-g^{7}-PDAAm$_{50}$ and PVA$_{181}$-g^{7}-PDAAm$_{100}$ nano-objects and (B) histograms of size distribution along with calculated average diameter values measured from particle analysis based on cryo-TEM images. In each case, at least 50 particles were analysed.

Additional Ice Growth data/Images.

Saline (0.01 M NaCl) was used in place of PBS in this work, due to the colloidal stability challenges (as discussed in the text). This low salt condition has been previously used to study colloids[7] and antifreeze proteins[8] and is sufficient to prevent false positives which occur when pure water is used alone.[9] Example control (no additive) ice wafers after 30 minutes annealing for PBS and NaCl are show below. Salts can have a significant impact on IRI and the magnitude of effect of any additive may be impacted by this which should always be considered.[10]

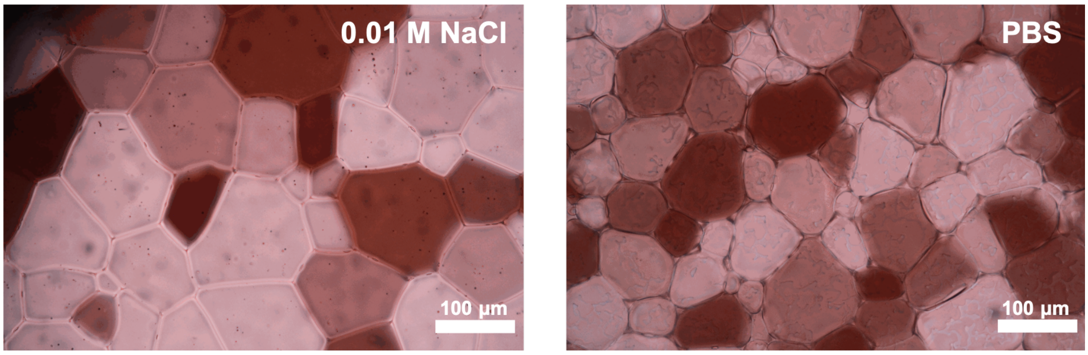

Figure S10. Cryomicrographs of ice crystal wafers of 0.01M NaCl and PBS controls.

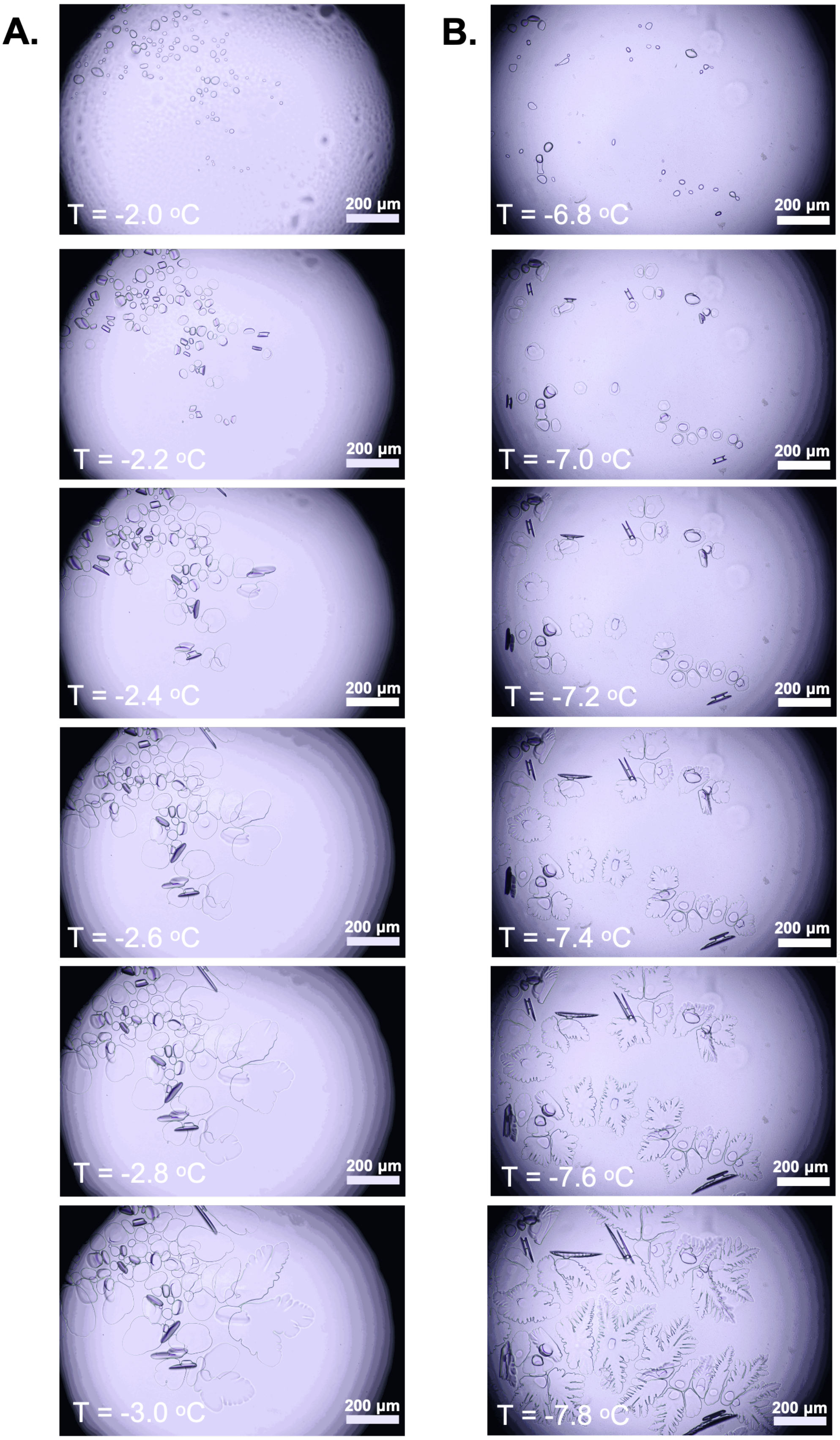

Figure S11. Modified sucrose sandwich ice shaping assay images for sucrose solution alone (A) and 1 mg.mL^{-1} of PVA$_{181}$-g^{7}-PDAAm$_{300}$ nanoparticles (B). Needle-shaped crystals are flat disks lying on their side rather than floating, with their round basal plane uppermost.

References

1 M. M. Tomczak, C. B. Marshall, J. A. Gilbert and P. L. Davies, *Biochem. Biophys. Res. Commun.*, 2003, **311**, 1041–1046.

2 M. Smallwood, D. Worrall, L. Byass, L. Elias, D. Ashford, C. J. Doucet, C. Holt, J. Telford, P. Lillford and D. J. Bowles, *Biochem. J.*, 1999, **340**, 385–391.

3 K. D. Kumble, J. Demmer, S. Fish, C. Hall, S. Corrales, A. DeAth, C. Elton, R. Prestidge, S. Luxmanan, C. J. Marshall and D. A. Wharton, *Cryobiology*, 2008, **57**, 263–268.

4 R. N. Johnson, R. S. Burke, A. J. Convertine, A. S. Hoffman, P. S. Stayton and S. H. Pun, *Biomacromolecules*, 2010, **11**, 3007–3013.

5 L. D. Blackman, K. E. B. Doncom, M. I. Gibson and R. K. O'Reilly, *Polym. Chem.*, 2017, **8**, 2860–2871.

6 L. D. Blackman, S. Varlas, M. C. Arno, Z. H. Houston, N. L. Fletcher, K. J. Thurecht, M. Hasan, M. I. Gibson and R. K. O'Reilly, *ACS Cent. Sci.*, 2018, **4**, 718–723.

7 T. Li, Y. Zhao, Q. Zhong and T. Wu, *Biomacromolecules*, 2019, **20**, 1667–1674.

8 C. A. Knight, D. Wen and R. A. Laursen, *Cryobiology*, 1995, **32**, 23–34.

9 C. I. Biggs, C. Stubbs, B. Graham, A. E. R. Fayter, M. Hasan and M. I. Gibson, *Macromol. Biosci.*, 2019, **19**, 1900082.

10 R. Surís-Valls and I. K. Voets, *Biomolecules*, 2019, **9**, 347.
